# The First Nickelacarborane with *closo*-*nido* Structure

**DOI:** 10.3390/molecules25246009

**Published:** 2020-12-18

**Authors:** Ekaterina P. Andreichuk, Sergey A. Anufriev, Kyrill Yu. Suponitsky, Igor B. Sivaev

**Affiliations:** 1 A.N. Nesmeyanov Institute of Organoelement Compounds, Russian Academy of Sciences, 28 Vavilov Street, 119991 Moscow, Russia; katenino16@gmail.com (E.P.A.); trueman476@mail.ru (S.A.A.); kirshik@yahoo.com (K.Y.S.); 2 Higher Chemical College at the Russian Academy of Sciences, D.I. Mendeleev Russian Chemical Technological University, 9 Miusskaya Square, 125047 Moscow, Russia; 3 N.S. Kurnakov Institute of General and Inorganic Chemistry, Russian Academy of Sciences, 31 Leninskii Avenue, 119991 Moscow, Russia

**Keywords:** nickelacarboranes, nickel bis(dicarbollide), pyridine derivative, polyhedral contraction, single crystal X-ray diffraction

## Abstract

The first nickelacarborane with *closo-nido* structure [10′,11′-(Py)_2_-3,9′-Ni(1,2-C_2_B_9_H_11_)(7′,8′-C_2_B_8_H_8_)] was isolated from the reaction of nickel(IV) bis(dicarbollide) with pyridine. The molecular structure of this complex was determined by single crystal X-ray diffraction. The nickel atom is a common vertex for the *closo*-NiC_2_B_9_ cluster and the *nido*-NC_2_B_8_ cluster where it is located together with carbon atoms in the open NiC_2_B_2_ pentagonal face. It is assumed that its formation proceeds through the nucleophile-induced removal of the B(6)H vertex followed by rearrangement of the forming 11-vertex cluster, which most likely proceeds through a sequence of closing and opening reactions.

## 1. Introduction

Due to the practically unlimited possibility of varying the structure of carboranes, including by replacing hydrogen atoms with various groups, transition metal complexes with carborane ligands are one of the most exciting areas of modern chemistry [1,2,3]. In addition to purely academic interest, a number of them show excellent prospects for practical use. Thus, nickel bis(dicarbollide) and its derivatives [4] attract interest of researchers due to their potential applications in materials science, including molecular switches [5,6,7,8], solar cells [9,10] and conductive metal-organic frameworks [11]. At the same time, the available information on the stability of these complexes is fragmentary and rather contradictory [12,13,14,15]. This prompted us to study the stability of nickel(IV) bis(dicarbollide) under various conditions. Earlier, we found that boiling nickel bis(dicarbollide) in ethanol leads to its decomposition with the formation of a mixture of *nido*-carborane and its 3-ethoxy derivative as the main products [16].

In this contribution we report the formation of the first nickelacarborane with a *closo-nido* structure in the reaction of nickel(IV) bis(dicarbollide) with boiling pyridine.

## 2. Results and Discussion

**Scheme 1:**
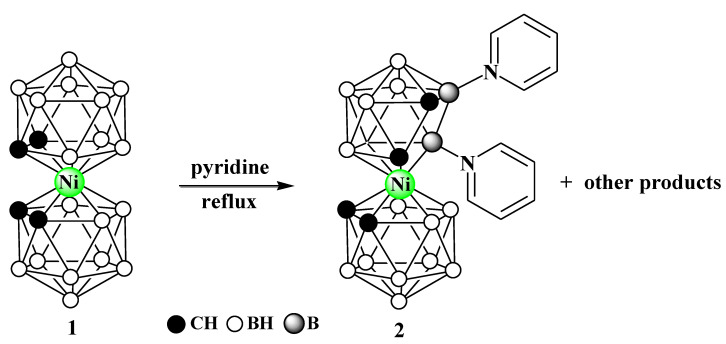
Reaction of nickel(IV) bis(dicarbollide) with pyridine.

It was found that prolonged refluxing nickel(IV) bis(dicarbollide) (**1**) in pyridine leads to its complete transformation into a mixture of various nickelacarboranes, mainly of paramagnetic nature. The separation of this mixture by column chromatography on silica gave a diamagnetic nickelacarborane [10′,11′-(Py)_2_-3,9′-Ni(1,2-C_2_B_9_H_11_)(7′,8′-C_2_B_8_H_8_)] (**2**), the structure of which was established by single crystal X-ray diffraction. The reaction is characterized by good reproducibility with an isolated yield of **2** ranging from 19 to 23% (Scheme 1).

The ^1^H NMR spectrum of **2** contains a set of signals from two different *N*-substituted pyridines in the range of 8.9–7.6 ppm and four signals from different CH groups of carborane ligands at 3.55, 3.33, 2.80 and 1.87 ppm, while the ^11^B NMR spectrum contains two sets of signals corresponding to symmetrical and asymmetrical carborane ligands (See Supplementary Materials). It should be noted that determination of the structure of metallacarboranes containing carborane ligands of different geometry is a difficult task that normally cannot be solved using methods of NMR spectroscopy due to signal overlap and, therefore, requires single crystal X-ray diffraction study [17,18,19,20,21,22].

**Figure 1:**
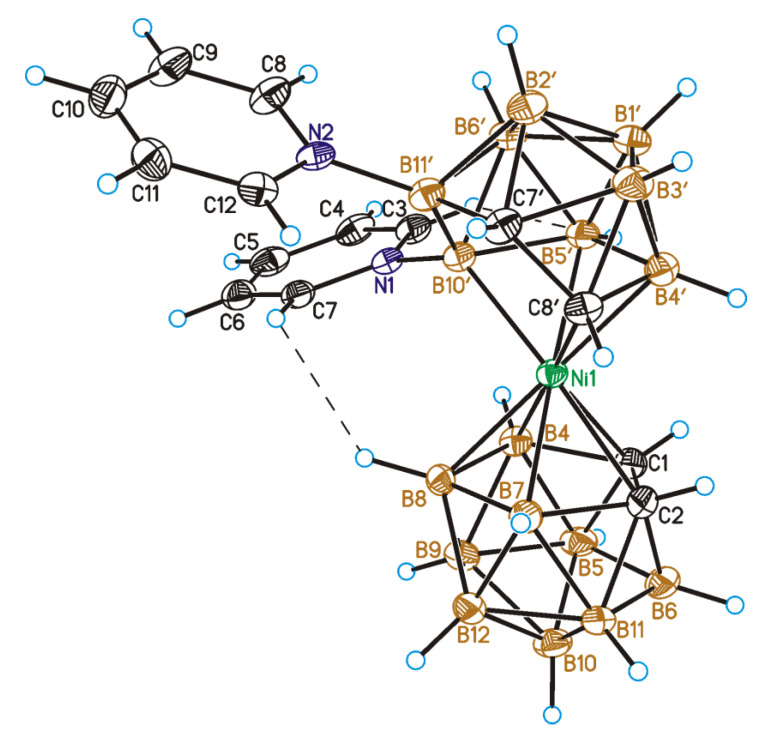
General view of complex **2** showing atomic numbering intramolecular CH⋯HB contacts. Thermal ellipsoids are drawn at 50% probability level.

An asymmetric unit cell of the complex **2** contains one molecule (Figure 1). In the structure of the complex, the nickel atom is a common vertex for the *closo*-NiC_2_B_9_ cluster and the *nido*-NC_2_B_8_ cluster, where it is located together with carbon atoms in the open NiC_2_B_2_ pentagonal face. Each of the remaining two boron atoms on the pentagonal face is bonded to a pyridine molecule.

The Ni-B bonds in the *nido*-fragment (2.093(3)–2.119(3) Å) are only slightly shorter than in the *closo*-fragment (2.103(2)–2.127(3) Å), while the Ni-C bond lengths differ more significantly (2.002(2) and 2.105(2)–2.170(2) Å for the *nido*- and *closo*-fragments, respectively). The same trend is observed also for the C-C bond lengths (1.508(3) and 1.573(3) Å for the *nido*- and *closo*-fragments, respectively). The *B*-*N* lengths equal 1.553(3) and 1.561(3) Å, that are somewhat shorter than in a related cobaltacarborane (Et_4_N)[11′-Py-3,9′-Co(1,2-C_2_B_9_H_11_)(7′,8′-C_2_B_8_H_10_)] (1.592(11) Å) [23], but close to those found in pyridinium derivatives of *nido*-carborane [9-Py-7,8-C_2_B_9_H_11_] (1.546(2) Å) [24] and metallacarboranes [4-Py-3-(C_4_Me_4_)-3,1,2-CoC_2_B_9_H_10_] (1.548(3) Å) [24], [8-Py-3,3′-Co(1,2-C_2_B_9_H_10_)(1′,2′-C_2_B_9_H_11_)] (1.556(5) Å) [25], [8-Py-3,3′-Fe(1,2-C_2_B_9_H_10_)(1′,2′-C_2_B_9_H_11_)] (1.548(6) and 1.553(6) Å) [25]. The relative orientation of the carborane ligands can be described by the pseudotorsion angle C1 Centroid (C1-C2-B7-B8-B4) Ni1-B10’ that is equal to 104.0 (2)°. Slightly shortened intramolecular CH⋯HB contacts were found between the pyridine substituent closest to the metal atom and the dicarbollide ligands. To assess their possible contribution to the stabilization of the observed conformation of complex **2**, we carried out its QTAIM (quantum theory of atoms in molecules) study [26,27]. Energy of intramolecular noncovalent interactions was estimated based on their correlation with the energy density function [28] that was found to be reliable for different types of weak noncovalent interactions [29,30,31]. Two CH⋯HB noncovalent contacts (shown in Figure 1) were observed (B5’-H5’⋯H3A-C3, 2.40Å, −2.4 kcal/mol; B8-H8⋯H7A-C7, 2.31Å, −2.6 kcal/mol).

It should be noted that neither other transition metal bis(dicarbollide) complexes, nor *ortho*-carborane itself, do not undergo deboronation with pyridine, whereas *C-*halogen derivatives were found to be accessible for nucleophilic attack of pyridine and its derivatives [32]. Complex **2** is the first example of a nickelacarborane in which the metal atom is common to both the *closo*-icosahedral cluster and the *nido*-cluster formed by removing one vertex from the icosahedron. A related cobaltacarborane [11′-Py-3,9′-Co(1,2-C_2_B_9_H_11_)(7′,8′-C_2_B_8_H_10_)]^−^ (**3**) was earlier prepared by removing one BH vertex on heating the parent cobalt bis(dicarbollide) [3,3′-Co(1,2-C_2_B_9_H_11_)_2_]^−^ in a 25% aqueous solution of potassium hydroxide at 95 °C, followed by the oxidation of the formed *nido*- structure to the *closo-* one with 30% hydrogen peroxide, and the reaction with pyridine, which leads to the opening of the 11-vertex metallacarborane the substitution of one hydrogen atom with pyridine [33,34]. In general, the formation of complex **2** can be described as a polyhedral contraction reaction [33,35,36].

Taking into account that the BH group at position 6 is the most susceptible to nucleophilic attack in the 3,1,2-MC_2_B_9_ metallacarboranes, we believe the formation of complex **2** should also proceed through the removal of this vertex followed by rearrangement of the 11-vertex cluster, which most likely proceeds through a sequence of closing and opening reactions, rather than via detachment and reattachment of the carborane ligand. Therefore, it is reasonable to assume that other formed nickelacarboranes are related to cobaltacarboranes, which are intermediate products of the formation of complex **3**. The identification of these products is in progress.

## 3. Materials and Methods

### 3.1. General Methods

Nickel(IV) bis(dicarbollide) (**1**) was prepared according to the published procedure [37]. The reaction progress was monitored by thin layer chromatography (Merck F254 silica gel on aluminum plates) and visualized using 0.5% PdCl_2_ in 1% HCl in aq. MeOH (1:10). Acros Organics silica gel (0.060–0.200 mm) was used for column chromatography. The NMR spectra at 400 MHz (^1^H), 128 MHz (^11^B) and 100 MHz (^13^C) in acetone-*d*_6_ were recorded with a Varian Inova 400 spectrometer. The residual signal of the NMR solvent relative to tetramethylsilane was taken as the internal reference for ^1^H and ^13^C NMR spectra. ^11^B NMR spectra were referenced using BF_3_·Et_2_O as an external standard.

The X-ray diffraction experiment for compound **2** was carried out using a SMART APEX2 CCD diffractometer (λ(Mo-Kα) = 0.71073 Å, graphite monochromator, *ω*-scans) at 120 K. Collected data were processed by the SAINT and SADABS programs incorporated into the APEX2 program package [38]. The structure was solved by direct methods and refined by the full-matrix least-squares procedure against *F*^2^ in anisotropic approximation. The refinement was carried out with the SHELXTL program [39]. The CCDC number (2046492) contain the supplementary crystallographic data for this paper. These data can be obtained free of charge via www.ccdc.cam.ac.uk/data_request/cif.

### 3.2. Reaction of Nickel(IV) Bis(Dicarbollide) with Pyridine

A solution of nickel(IV) bis(dicarbollide) (50 mg, 0.3 mmol) in 30 mL of pyridine was stirred under reflux for 50 h. Thereafter, solution was cooled to room temperature and concentrated under reduced pressure. The crude product was subjected to column chromatography on silica using of dichloromethane as eluent. The third boron-containing fraction gave dark crystals of **2** (17 mg, yield 23%). ^1^H NMR: δ 8.89 (d, 2H, J = 7.0 Hz, *o*-C*H*_Py_), 8.70 (2H, d, J = 6.8 Hz, *o*-C*H*_Py_), 8.46 (1H, t, J = 7.0 Hz, *p*-C*H*_Py_), 8.19 (1H, t, J = 6.8 Hz, *p*-C*H*_Py_), 8.01 (2H, t, J = 7.0 Hz, *m*-C*H*_Py_), 7.60 (2H, t, J = 6.8 Hz, *m*-C*H*_Py_), 4.0–0.4 (15H, br, B*H*_Carb_), 3.55 (1H, s, C*H*_Carb_), 3.33 (1H, s, C*H*_Carb_), 2.80 (1H, s, C*H*_Carb_), 1.87 (1H, s, C*H*_Carb_) ppm; ^11^B NMR: δ 15.2 (1B, s, *B*-*N*), 7.7 (1B, d, J = 116 Hz), 2.3 (1B, d, J = 149 Hz), 0.8 (1B, s, *B*-*N*), –2.1 (1B, d, J = 146 Hz), –4.6 (2B, d, J = 137 Hz), –6.4 (2B, d, J = 146 Hz), –7.8 (1B, d, J = 114 Hz), –10.9 (1B, d, J = 133 Hz,), –17.4 (3B, d, J = 138 Hz), –20.2 (1B, d, J = 163 Hz), –22.9 (1B, d, J = 163 Hz), –31.6 (1B, d, J = 139 Hz) ppm; ^13^C NMR: δ 149.8 (*o*-*C*H_Py_), 146.6 (*o*-*C*H_Py_), 143.5 (*p*-*C*H_Py_), 143.1 (*p*-*C*H_Py_), 128.6 (*m*-*C*H_Py_), 126.1 (*m*-*C*H_Py_), 54.1 (*C*H_Carb_), 45.7 (*C*H_Carb_), 40.9 (*C*H_Carb_), 40.2 (*C*H_Carb_) ppm. *Crystallographic data:* C_14_H_29_B_17_N_2_Ni are monoclinic, space group *P*2_1_/*n*: *a* = 13.0440(8) Å, *b* = 9.8669(6) Å, *c* = 18.6798(11) Å, *β*= 98.2660(10)°, *V* = 2379.2(2) Å^3^, *Z* = 4, *M* = 467.87, *d*_cryst_ = 1.306 g·cm^−3^. *wR*2 = 0.0990 calculated on *F*^2^*_hkl_* for all 6244 independent reflections with 2*θ* < 56.4°, (*GOF* = 1.020, *R* = 0.0408 calculated on *F_hkl_* for 4310 reflections with *I* > 2*σ*(*I*)).

## Supplementary Materials

The following are available online, NMR spectra of compound **2**.
