# Supplementary material for: The First Nickelacarborane with closo-nido Structure
Source: Molecules. 2020 Dec 18;25(24):6009. doi: 10.3390/molecules25246009 (PMC7767031; doi:10.3390/molecules25246009)
Supplement: Supplementary file 1 [file molecules-25-06009-s001.zip › Molecules-SI.pdf]

SUPPORTING INFORMATION

The First Nickelacarborane with *closo-nido* Structure

Ekaterina P. Andreichuk [1,2], Sergey A. Anufriev [1], Kyrill Yu. Suponitsky [1,3] and Igor B. Sivaev [1,*]

[1] A.N. Nesmeyanov Institute of Organoelement Compounds, Russian Academy of Sciences, 28 Vavilov Str., 119991, Moscow, Russia; katenino16@gmail.ru (E.P.A.); trueman476@mail.ru (S.A.A.); kirshik@yahoo.com (K.Y.S.)

[2] D.I. Mendeleev Russian Chemical Technological University, 9 Miusskaya Sq., 125047, Moscow, Russia

[3] N.S. Kurnakov Institute of General and Inorganic Chemistry, Russian Academy of Sciences, 31 Leninskii Av., 119991, Moscow, Russia

* Correspondence: sivaev@ineos.ac.ru; Tel.: +7-916-590-2025

NMR spectra of compound **2** **2**

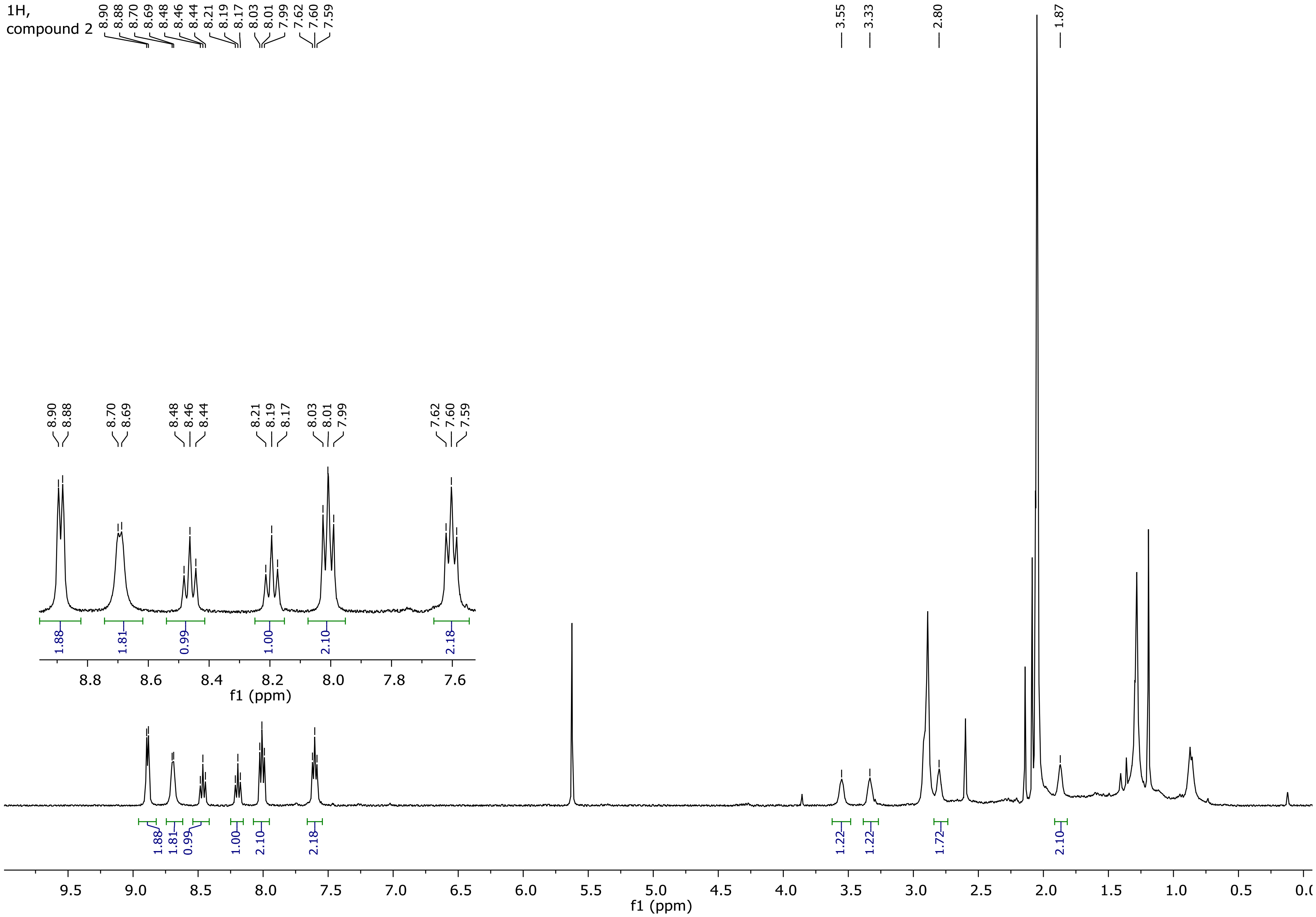
1H,
compound 2
8.90
8.88
8.70
8.69
8.48
8.46
8.44
8.21
8.19
8.17
8.03
8.01
7.99
7.62
7.60
7.59
3.55
3.33
2.80
1.87
1.88
1.81
0.99
1.00
2.10
2.18
1.22
1.22
1.72
2.10
f1 (ppm)
f1 (ppm)

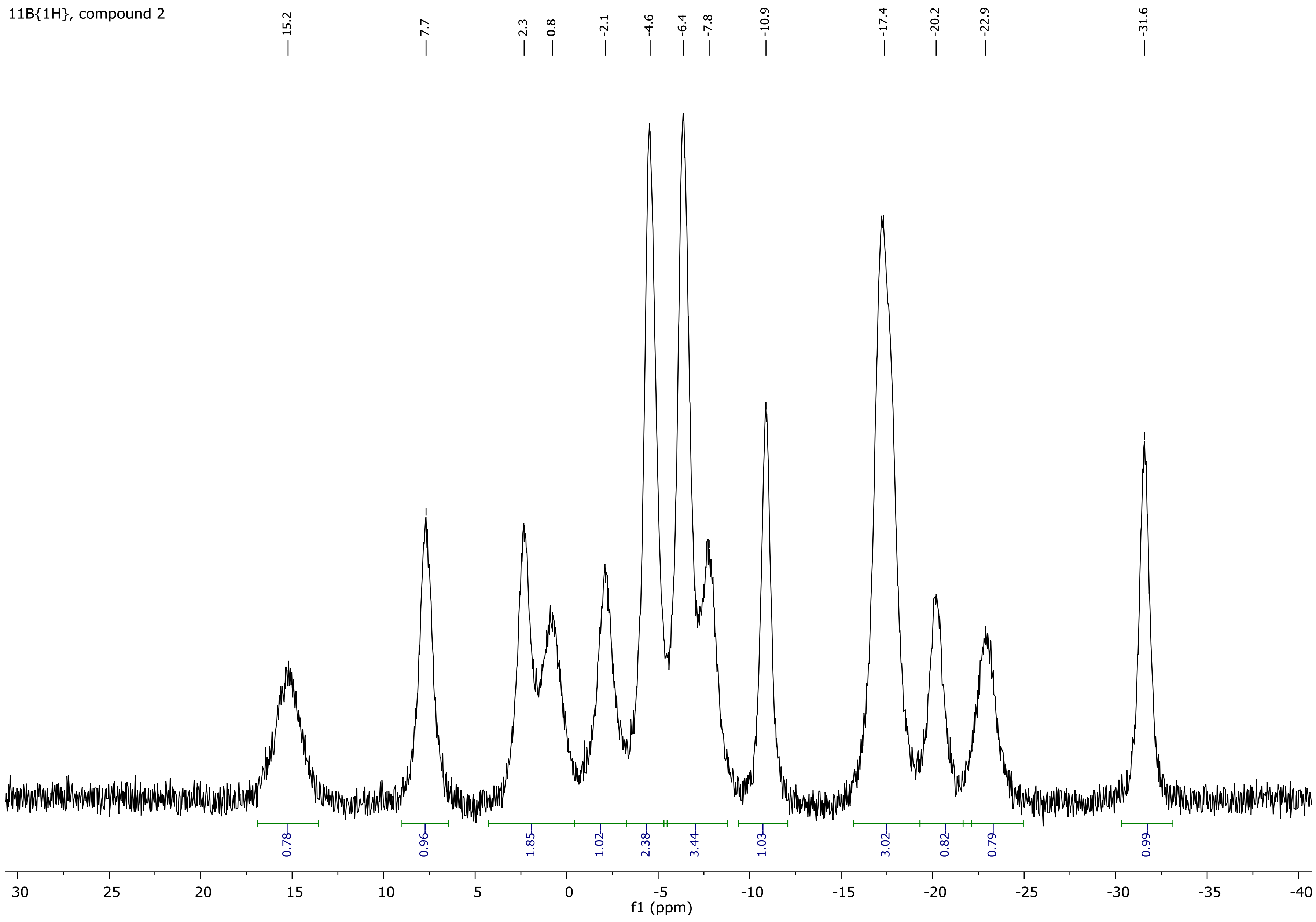
11B{1H}, compound 2
15.2
7.7
2.3
0.8
-2.1
-4.6
-6.4
-7.8
-10.9
-17.4
-20.2
-22.9
-31.6
0.78
0.96
1.85
1.02
2.38
3.44
1.03
3.02
0.82
0.79
0.99
30
25
20
15
10
5
0
-5
-10
-15
-20
-25
-30
-35
-40
f1 (ppm)

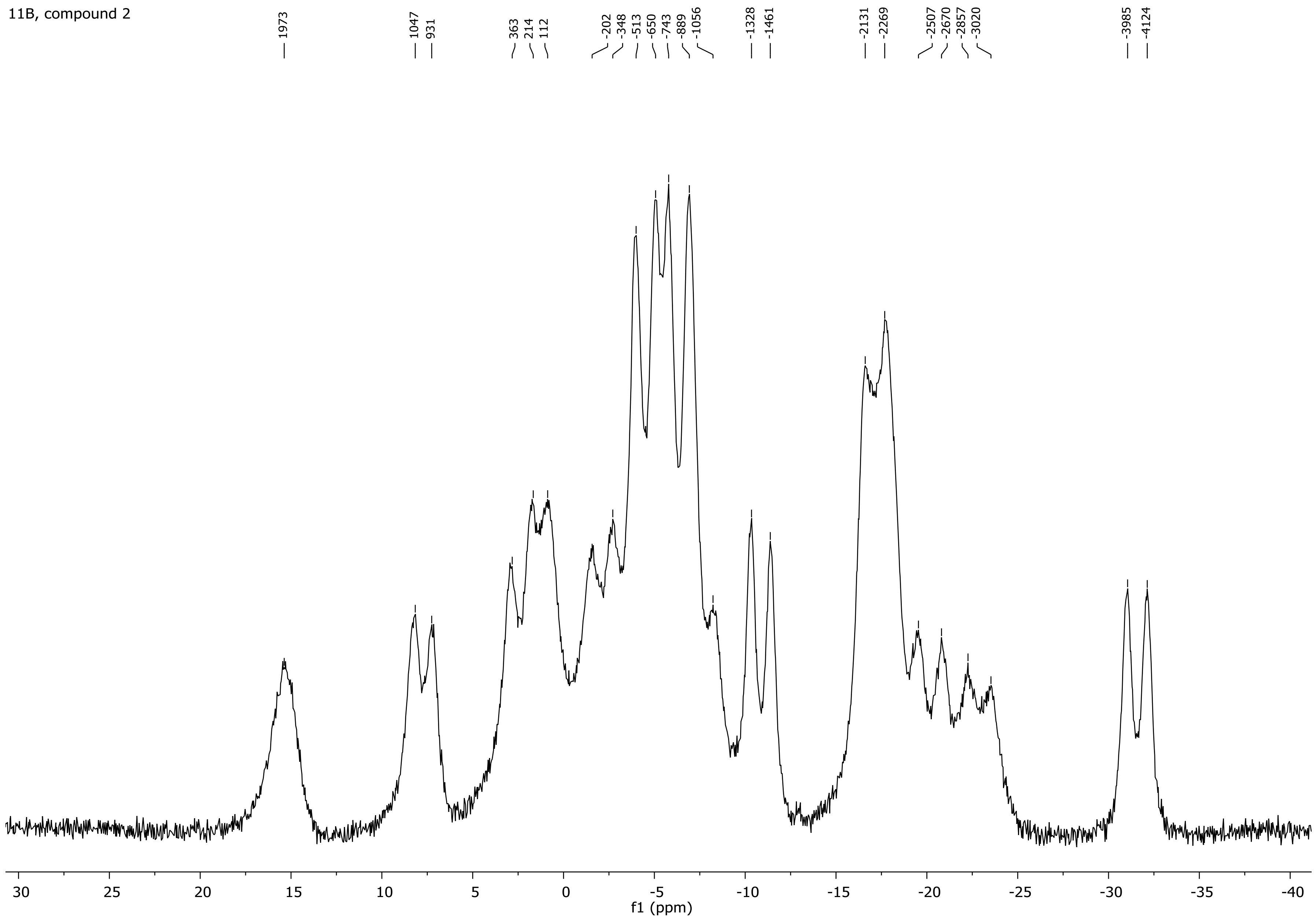
11B, compound 2
1973
1047
931
363
214
112
-202
-348
-513
-650
-743
-889
-1056
-1328
-1461
-2131
-2269
-2507
-2670
-2857
-3020
-3985
-4124
30
25
20
15
10
5
0
-5
-10
-15
-20
-25
-30
-35
-40
f1 (ppm)

13C{1H}, compound 2

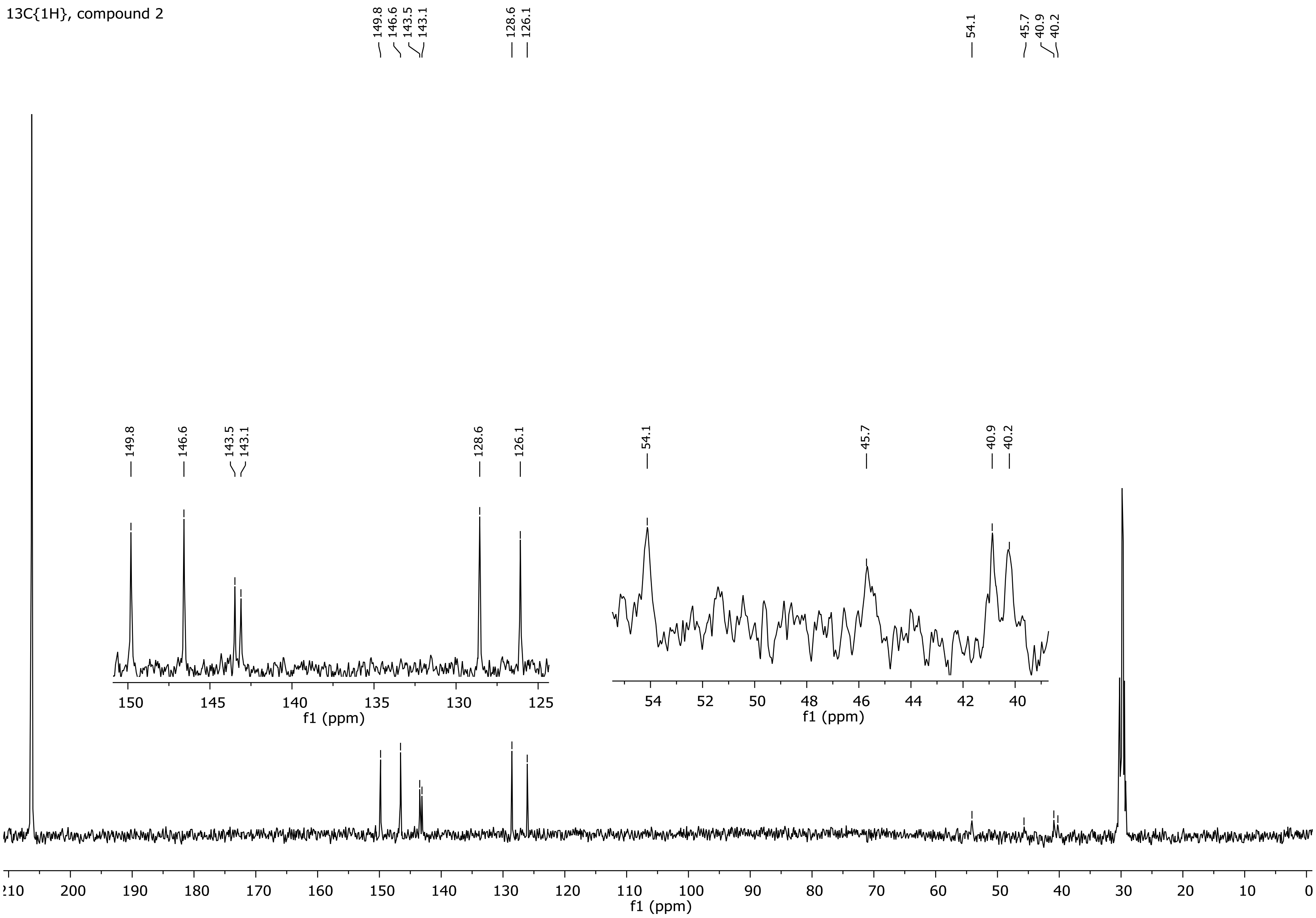

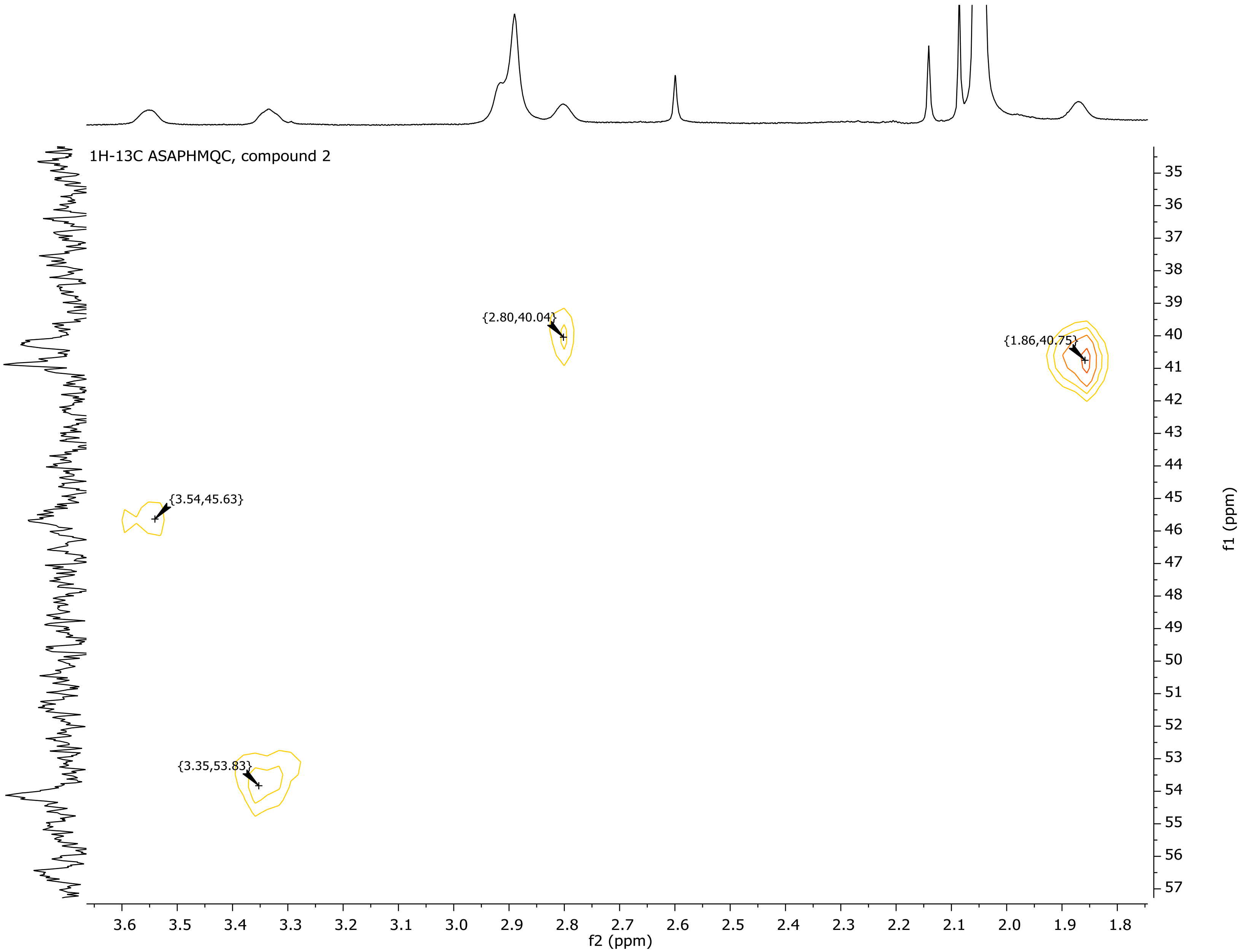

1H-13C ASAPHMQC, compound 2
{2.80,40.04}
{1.86,40.75}
{3.54,45.63}
{3.35,53.83}
f1 (ppm)
f2 (ppm)
